# Fisheye piezo polymer detector for scanning optoacoustic angiography of experimental neoplasms

**DOI:** 10.1016/j.pacs.2023.100507

**Published:** 2023-05-07

**Authors:** Alexey Kurnikov, Grigory Volkov, Anna Orlova, Andrey Kovalchuk, Yulia Khochenkova, Daniel Razansky, Pavel Subochev

**Affiliations:** a Institute of Applied Physics, Russian Academy of Sciences, 46 Ulyanov Str., Nizhny Novgorod 603950, Russia; b National Medical Research Center of Oncology named after N. N. Blokhin, Kashirskoe highway 23, Moscow 115522, Russia; c Institute of Pharmacology and Toxicology, Faculty of Medicine, UZH Zurich, Rämistrasse 71, Zurich 8006, Switzerland; d Institute for Biomedical Engineering, Department of Information Technology and Electrical Engineering, ETH Zurich, Gloriastrasse 35, Zurich 8092, Switzerland

**Keywords:** Optoacoustic mesoscopy, Experimental neoplasms, Limited view, Optoacoustics, Photoacoustics, Biophotonics, Numerical aperture, Full angular coverage, Piezopolymer, Broadband ultrasound detectors

## Abstract

A number of optoacoustic (or photoacoustic) microscopy and mesoscopy techniques have successfully been employed for non-invasive tumor angiography. However, accurate rendering of tortuous and multidirectional neoplastic vessels is commonly hindered by the limited aperture size, narrow bandwidth and insufficient angular coverage of commercially available ultrasound transducers. We exploited the excellent flexibility and elasticity of a piezo polymer (PVDF) material to devise a fisheye-shape ultrasound detector with a high numerical aperture of 0.9, wide 1–30 MHz detection bandwidth and 27 mm diameter aperture suitable for imaging tumors of various size. We show theoretically and experimentally that the wide detector’s view-angle and bandwidth are paramount for achieving a detailed visualization of the intricate arbitrarily-oriented neovasculature in experimental tumors. The developed approach is shown to be well adapted to the tasks of experimental oncology thus allows to better exploit the angiographic potential of optoacoustics.

## 1 Introduction

In clinical oncology, vascular features of neoplasms are actively studied to determine more accurate diagnostic criteria, develop new prognostic factors, and evaluate effectiveness of potential therapies. Likewise, preclinical studies into neovascular patterns are necessary for monitoring the disease progression and analyzing treatment responses [1]. Human or animal tumor cell lines can be employed for studying the development of malignant neoplasms in vivo. Experimental tumor inoculation is carried out by subcutaneous or orthotopic implantation of cells or other tumor material [2]. Although orthotopic tumors exhibit higher vascularity [3], subcutaneously growing tumors remain the most convenient model for the growth and investigation of malignant neoplasms, greatly facilitating the measurement of the nodal size [4] and allowing for the use of a number of imaging methods with a limited depth of diagnosis [5].

Optoacoustic angiography is among the most effective methods for label-free visualization of blood vessels including neovascular networks in tumor nodes [6]. Using short optical pulses and broadband ultrasonic receivers, this method makes it possible to obtain high-contrast 3D images of vascular networks with a spatial resolution of several tens of microns at a depth of several millimeters in optical dense tissues [7]. Recently, the method has been widely used both for clinical diagnosis of superficially located tumors [8] and for the study of vascular patterns during and after treatment in preclinical tumor models [9], [10].

Even though the technical implementations of the developed OA systems and image reconstruction algorithms have greatly advanced over the recent years, a number of fundamental problems remain that hinder anatomically accurate rendering of arbitrarily-oriented vascular networks in 3D. One major problem is the limited view of the ultrasonic detectors, which prevents registration of acoustic waves propagating at a steep angle to the transducer surface or the scanning plane. In optoacoustic tomography (OAT) systems, a number of hardware- and software-based solutions have been proposed. For instance, the use of a linear array in combination with a reflector [11] or two linear arrays with adjustable orientation [12] makes it possible to significantly increase the effective detection area and tomographic coverage, thereby improving the reconstructed image quality. Also, the use of advanced reconstruction algorithms and deep learning networks can aid in mitigating common image artifacts associated with a limited field of view, both for cross-sectional and volumetric OAT systems [13], [14], [15].

For monitoring subcutaneously grafted tumors, scanning acoustic-resolution optoacoustic mesoscopy (OAM) systems are more commonly used, where the detection area is highly dependent on the numerical aperture (NA) of the single-element ultrasonic transducer. It has previously been shown that image quality can markedly be improved when using detectors or scanning geometries with higher angular coverage, resulting in more continuous vascular network renderings [16], [17]. By increasing the acoustic collection angle, the system can better depict arbitrarily-oriented structures emitting optoacoustic signals with strong directivity.

In this work, to reduce the influence of the limited field of view, an OAM system has been developed using a detector with an ultrahigh NA of 0.9 based on a flexible piezo polymer PVDF film. The material used to develop the PVDF detector has a number of significant advantages. First, it exhibits a broadband frequency response enabling simultaneous visualization of vessels across a wide range of dimensions [18]. Secondly, its acoustic impedance is close to water, hence the signal can be recorded with a sufficiently high SNR without the use of additional matching layers. Lastly, owing to the exceptional flexibility and elasticity of the material, very high NA can be accomplished. To demonstrate the feasibility of such a system, spiral phantom imaging and in vivo imaging of a subcutaneously grafted experimental mouse tumor were performed. The developed fish-eye detector with a focal length F = 15 mm, aperture A = 27 mm and an angle of view of 128° made it possible to cover a large part of the studied tumor (F-number of f/0.6), while showing superior image quality compared to a detector with standard angular coverage (F-number of f/1.3).

## 2 Materials and methods

### 2.1 Modeling ultrasound signals from extended sources

To demonstrate the challenging problem of limited view in OAM, we first performed a two-dimensional simulation of B-scan images obtained by scanning an object containing elongated optoacoustic sources stretching in multiple directions with a focused detector. The simulation was carried out in the MATLAB software environment using the k-Wave package [19]. The size of the two-dimensional image grid was 5 mm × 5 mm and the grid step was 25 µm. The medium was acoustically homogeneous water with an average sound speed of 1500 m/s and signal damping at the grid boundary. The detector had a radius of 2 mm and was focused on the source of ultrasonic signals in the center of the simulated area. The source consisted of five 1 mm long lines, oriented at different angles with respect to the scanning axis (0°, 30°, 60°) and two arcs with a curvature radius of 0.75 mm and central angles of 90° and 180°. The thickness of lines and arcs was equal to the grid spacing. To obtain an image, the source was moved with a step of 25 µm along the horizontal (X) axis in the range of 2 mm from the center of the modeled area. The images were obtained for detectors with different NA of 0.3, 0.7, and 1. The corresponding angular coverage of the detectors were 35°, 90°, and 180°. All obtained 2D data were reconstructed in the frequency domain with delay and sum method [20].

### 2.2 Optoacoustic imaging system

**Fig. 1:**
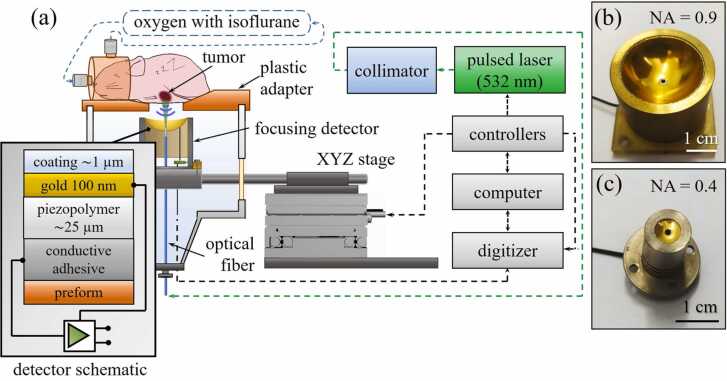
The developed scanning optoacoustic mesoscopy (OAM) system for imaging intricate arbitrarily-oriented neovascular network in experimental neoplasms. (a) System schematics. Two detector configurations based on PVDF piezo polymer film were evaluated with low (b) and high (c) numerical aperture (NA).

Visualization of the phantom and the experimental tumor was then carried out using customized OAM system (Fig. 1a). A diode-pumped laser ONDA532 (Bright Solutions, Italy) operating at 532 nm wavelength with 2 ns pulse duration, 0.5 mJ per-pulse energy and up to 100 kHz pulse repetition frequency (PRF) was used. The actual PRF of the laser during scanning was determined by the frequency of triggering sync pulses from the motion controller, thereby determining the effective scanning step along the fast moving scanning axis. Laser radiation was delivered to the target using multimode optical fiber FG550LEC (Thorlabs, USA) protruded through a 1 mm diameter aperture in the ultrasonic detector. The ultrasonic signals generated by the object as a result of the absorption of short laser pulses were recorded by a broadband spherical ultrasonic detector based on a PVDF piezo polymer film (PolyK, USA). The electric signals detected by the piezofilm were sent to an impedance matching amplifier with a uniform gain in frequency band from 0.3 to 100 MHz, located within the same housing. The detector was fixed on the scanning module (Fig. 1a) and placed into the hermetic immersion chamber filled with distilled water. The object was visualized through the upper open face of the immersion chamber, through an adapter manufactured using photopolymer 3D printer Sonic Mighty 4 K (Phrozen, China). Scanning was carried out automatically along two orthogonal axes with linear stages V-408.132020 (PI micos, Germany) in the range of up to 25 mm along both axes and with a scanning step of up to 20 µm. The signals recorded by the detector were digitized by a 16-bit analog-to-digital converter CSE25216 (GaGe, USA) at 500 MHz sampling rate. The digitized signals were subsequently processed by a customized Matlab-based software with graphical user interface (GUI). To search for the scanning area of interest, a three-coordinate device (Standa, Lithuania) was used. The distance between the detector and the object of study was adjusted in the mode of continuous visualization of the central B-scan with a frame rate of 2 Hz.

Experimental studies were carried out using two spherically focused detectors with different NA of 0.9 and 0.375 (≈0.4) (Fig. 1b-c). Focal length F, aperture A, and angular coverage α and NA for each detector are specified in Table 1. The ultra-high NA (or fisheye) detector was made on the basis of a piezo polymer 25-micron PVDF film with gold-plated electrodes, and the low NA detector based on a PVDF-TrFE film with a thickness of 20 µm was also made with gold-plated electrodes. As shown in the subsequent experiments, even though a detector with a smaller focal length had a significantly better sensitivity, better images were obtained using the fisheye detector.

**Table 1:** Parameters of piezopolymer detectors used for experiments.

	T, µm	F, mm	A, mm	α, deg.	material
**Standard detector (NA=0.4)**	20	8	6	44	PVDF-TrFE
**Fish-eye detector (NA=0.9)**	25	15	27	128	PVDF

### 2.3 Phantom and in vivo experiments

In order to directly compare performance of the two detectors, phantom and in vivo experiments were conducted. The fixed scanning step of 20 µm along the X and Y axes and temporal sampling interval of 5 ns was used in each of the experiments. Comparable levels of light irradiance for each detector in each experiment were established by varying the laser pulse energy. However, the laser pulse energy did not surpass 100 μJ, and the maximum light irradiance was approximately 3 mJ/cm^2^, which adhered to the safe exposure levels (20 mJ/cm^2^) set by the American National Standards Institute (ANSI).

To evaluate spatial resolution, the initial phantom test utilized 2D scanning of a 50 µm (Cospheric LLC, USA) light-absorbing microsphere. A subsequent phantom experiment involved lateral scanning of 50 µm copper wires at varying depths, aimed at comparing the depth of field in the raw signal domain (DOF-raw) and reconstruction domain (DOF-recon). The capability of visualizing more intricate structures with fisheye detector was demonstrated in phantom experiments by imaging a section of a 1.4 mm diameter spiral with a thickness of 0.1 mm.

For the in vivo experiment, a tumor model based on the SN-12 C renal cell carcinoma cell line was inoculated subcutaneously on the outer side of the thigh in Balb/C nu/nu mice. To create a model, a suspension of tumor cells in serum-free medium RPMI-1640 (Gibco, USA) at a concentration of 2 × 107/ml of the medium was mixed with Matrigel (Corning, USA) at a 2:1 ratio and injected into the animal in a volume of 0.2 ml. OAM visualization of the neoplasm was performed on the 125th day of tumor growth, with its volume reaching 700 mm^3^. During the experiment, the animal was anesthetized on a Zoomed Minor Vet anesthetic-respiratory machine (Zoomed, Russia) with a mixture of 1.5% isoflurane (Laboratorios Karizoo, Spain) and 100% oxygen at a gas flow rate of 0.1 l/min. The supply of a mixture of oxygen with isoflurane was carried out through a breathing mask on a plastic adapter, as shown in the scheme of the experimental setup. Registration of ultrasonic signals was carried out directly through distilled water without applying ultrasonic gel to the tumor. Images of the experimental tumor for both detectors were obtained in the scanning range of 10 mm × 10 mm with a scanning step of 20 µm and a scanning time of about 5 min. In addition to the 2D delay and sum reconstruction in two orthogonal planes applied to the 3D data set [21], the CLAHE (Contrast limited adaptive histogram equalization) algorithm [22] was also applied to the maximum intensity projection (MIP) images. All work related to animals was carried out in accordance with the protocol of the ethics committee No. 04 P dated July 29, 2021 of the Federal State Budgetary Institution "National Medical Research Center of Oncology named after N.N. Blokhin" of the Ministry of Health of Russia.

## 3 Results and discussion

**Fig. 2:**
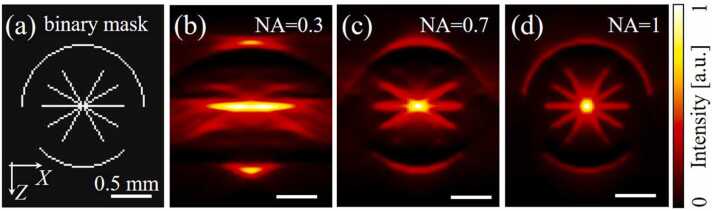
Modeling the detection of ultrasonic signals by detectors with different numerical aperture (NA) from a source containing absorbing structures propagating in arbitrary directions. (a) Binary mask of the simulated optoacoustic signal source. (b)-(d) Simulation results for three detectors with NA of 0.3, 0.7, and 1.

Fig. 2 shows a binary mask of the source of ultrasonic signals with simulation results for three detectors with a numerical aperture from 0.3 to 1. The image obtained for a detector with NA= 0.3 is very blurred, which is associated with both a degraded spatial resolution and a limited 35° effective angular coverage. Only a line parallel to the scanning axis and small parts of the arcs can be clearly visualized. On the other hand, the detector with NA= 0.7 exhibited better spatial resolution with both horizontal line sources and the ones inclined at ± 30° to the scanning axis as well as an entire arc with a smaller central angle of 90° clearly distinguishable in the images. However, line sources with ± 60° angle of inclination to the scanning axis are still invisible, while only a part of the arc with a large central angle of 180° could be rendered. The image obtained for the NA= 1 detector fully reproduces the original source configuration.

**Fig. 3:**
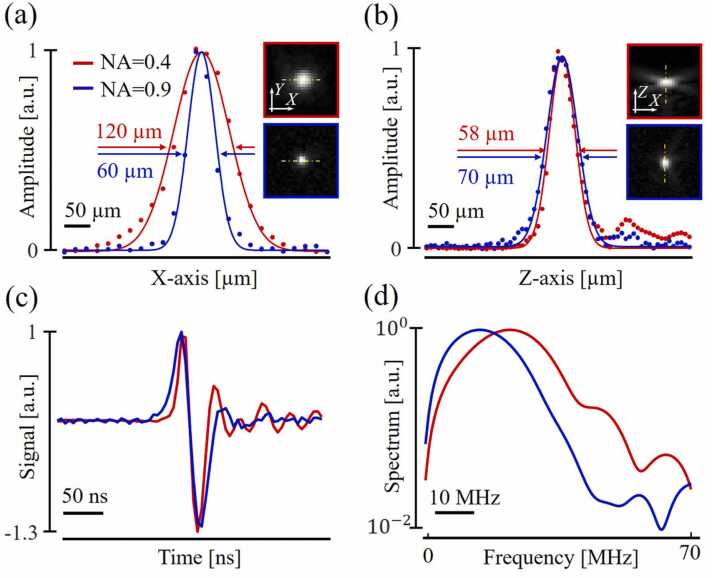
Experimental OA images of 50 µm diameter light-absorbing microsphere. (a) FWHM for reconstructed profile along the x-axis. (b) The corresponding profile of reconstructed OA signal along the z-axis. (c) and (d) are the corresponding raw OA signals and their spectra.

Fig. 3 represents the spatial resolution for the developed detectors estimated by scanning a black polystyrene microsphere phantom. The lateral/axial resolution (Fig. 3a-b) was defined as the full width at half maximum (FWHM) for the X-axis and Z-axis profiles passing through the center of the microsphere, and was measured to be 120/58 µm and 60/70 µm for detectors with NA of 0.4 and 0.9, respectively. Fig. 3(c-d) also shows the temporal signals from the microsphere and their spectra, evincing of the ultrawideband ultrasound detection properties of the PVDF material. The difference in the presented spectra can be associated with different thicknesses of the piezofilms (20/25 µm) and different focal lengths of the detectors (8/15 mm).

**Fig. 4:**
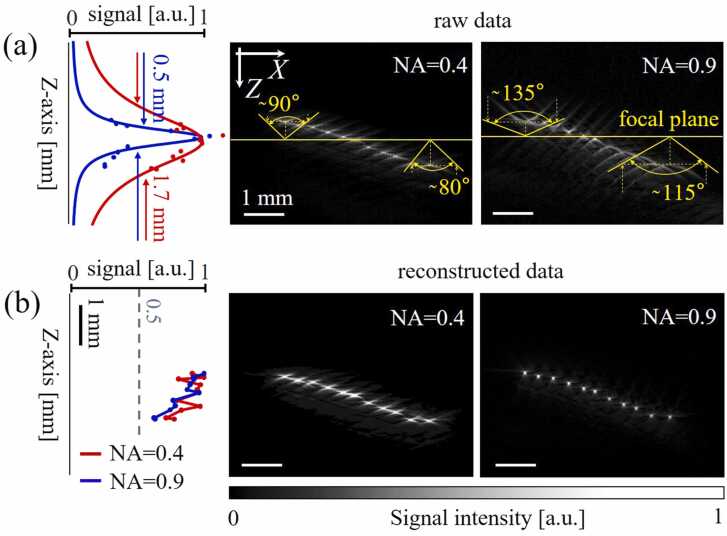
Imaging of 50 µm copper wires. (a) and (b) Raw B-scans with angles measured for the edge arcs, as well as signal profiles along the z axis with estimated DOF-raw values (0.5 mm for NA=0.9 and 1.7 mm for NA=0.4). (c) and (d) Reconstructed B-scans.

Fig. 4 shows the raw and reconstructed B-scans, with the profiles of local maxima plotted side by side along the z-axis. For the raw data, the DOF-raw evaluation considered signal values from the coordinates corresponding to the maxima in the reconstructed data, with a slight deviation at points falling on the intersection between the arcs. In Fig. 4a, the dots on the signal profiles mark the experimental data, whereas the solid curves indicate the approximation of these values by the Lorentz function. The DOF-raw value was measured as FWHM from the approximating curves and was 0.5 mm and 1.7 mm for detectors with NA of 0.9 and 0.4, respectively. Although the recorded signals quickly decrease in amplitude when moving out of the focal area of the high NA detector, the signal levels are mostly restored in the reconstructed images owing to the summation of signals over extended arcs. The amplitude profile of the reconstructed signals along the z axis is yet characterized by attenuation associated with scattering and absorption of optical and ultrasonic signals.

The effective view angle for the two detectors was also experimentally estimated from the raw B-scans. The results demonstrated that for the large NA detector objects outside the focal plane appear as extended arcs, which is not the case for the low NA detector. The angles were measured for the extreme upper and lower arcs from the focal plane and amounted to 135°/115° for the NA= 0.9 detector and 90°/80° for the NA= 0.4 detector. The experimentally measured values of the angle for the NA= 0.4 detector differ significantly from the angle specified by the fabricated geometry.

Fig. 4(b) demonstrates the signal profiles for the reconstructed data, which are characterized by a more uniform signal distribution for both detectors compared to the raw data.

Signals of different frequencies, collected by both detectors from different directions are further involved in acoustic reconstruction and make it possible to reconstruct sources that are physically located far beyond the DOF-raw levels (Fig. 4a). However, when comparing the signal (Fig. 4a) versus reconstruction (Fig. 4b) domains, it is the fish-eye detector benefiting the most from such DOF-recon extension. Nevertheless, the presented estimations of DOFs (Fig. 4) are relevant to just one possible imaging scenario with fixed source diameter and orientation.

**Fig. 5:**
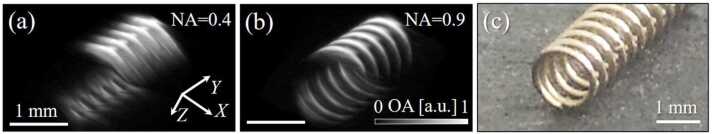
Reconstructed OA images of a spiral phantom obtained by (a) traditional detector with NA= 0.4 and (b) fish-eye detector with NA= 0.9. (c) Photography of the spiral phantom.

The ability of each detector to image a more complex spiral phantom representing all possible orientations of the sources with respect to the scanning plane is showcased in Fig. 5, aligning well with the numerical simulation results in Fig. 2. Results obtained with the fisheye detector (Fig. 5b) demonstrate nearly accurate visualization of the spiral turns. The absence of closure of the lower and upper arcs of the turns in the image was due to the incomplete 128° angular coverage and the corresponding decrease in the image contrast for extended angles. Also, the influence of optical and ultrasound shadowing cannot be ruled out. A photography of the investigated spiral is also shown for clarity (Fig. 5c).

**Fig. 6:**
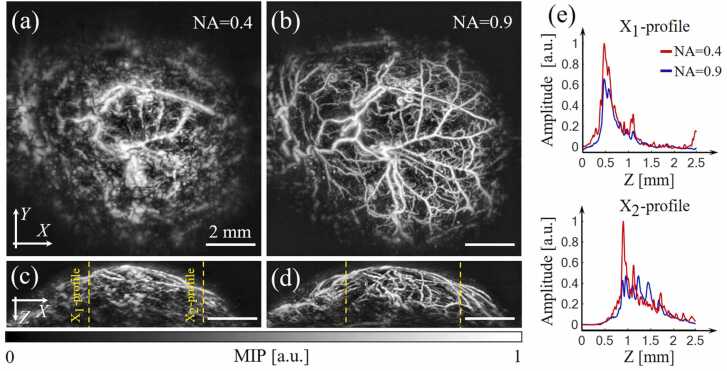
Imaging of neovascular network in an experimental SN-12 C tumor. (a) Top view of the image acquired with the NA = 0.4 detector. (b) The corresponding image for the NA= 0.9 detector. (c) and (d) are the corresponding side views. (e) One-dimensional signal profiles along the x axis, as denoted in panel (c).

Finally, the two detectors were used for in vivo imaging of a subcutaneously grafted mouse tumor. Fig. 6(a-d) show MIP images along the XY and XZ planes. The result obtained using a detector with NA= 0.9 (Fig. 6b,d) demonstrated a detailed and continuous image of the entire uninterrupted tumor vasculature patterns clearly discerning both the smallest tortuous capillaries as well as large extended blood vessels. On the other hand, vessels in the center of the imaged area had a blurry appearance in the images acquired with the NA= 0.4 detector further featuring separated discontinuous fragments in the periphery. The inability to visualize smallest capillaries with the low NA detector was associated with both poor resolution and high tortuosity of the vessels. Large inclined vessels extending from the center of the tumor to its periphery also partially disappeared from the image due to the poor angular coverage of the detector.

The observed effects can be mainly attributed to the fact that elongated vessel structures mainly emit highly directive radially diverging cylindrical ultrasonic waves. An ultrasonic detector with a small viewing angle is thus unable to efficiently detect waves propagating at significant angles to the scanning plane, which was also observed on individual signal profiles for each of the detectors (Fig. 6e). Note that the X_1_-profile (denoted in Fig. 6c) mainly crossed vessels parallel to the scanning plane, whereas the X_2_-profile extended through the vessels located at different angles to the same plane. As a result, while X_1_-profiles were similar for both detectors, the X_2_-profile for the small NA detector failed to detect a major portion of the generated signals.

The conducted numerical experiments did not involve optical modeling, whilst the phantom and in vivo experiments have employed a single optical fiber with a standard numerical aperture. Nevertheless, the amplitudes of optoacoustic signals are dependent on the local laser fluence. Hence, the diameter of the optical spot on the surface of biological tissue should be made wide enough in order to maximize benefits of the large numerical aperture detector for tomographic image reconstruction. Conversely, increasing the numerical aperture would inevitably result in diminished laser fluence thus stronger noise in the images. Therefore, the numerical aperture of the optical fiber should be optimized based on characteristics of the medium under study and ultrasonic detector properties.

## 4 Conclusions

PVDF material was selected owing to its excellent flexibility and elasticity, which made it possible to achieve ultrahigh numerical aperture of NA = 0.9. The developed OAM system using a wide 128° angle detector has demonstrated detailed visualization of arbitrarily-oriented vessels in experimental neoplasms. Compared to low NA detectors, the resulting tumor images manifested superior spatial resolution while providing more detailed information about structures oriented at significant angles to the scan plane. The ultrawideband frequency response of the piezo polymer material made it possible to visualize both the microvasculature of the tumor as well as larger vessels. The energy of laser pulses did not exceed the energy of the established standards for the safe use of laser radiation, which was nevertheless sufficient for detecting signals with a high SNR. Detection sensitivity can potentially be increased with PVDF copolymers, such as PVDF-TrFE, which has a larger piezoelectric modulus d_33_. Similar approaches can be attempted for mitigating limited view effects in OAT by means of PVDF-based hemispherical ultrasonic arrays as well as in optical-resolution optoacoustic microscopy systems employing coaxial illumination through transparent electrodes.

## Funding

Optoacoustic system development and phantom experiments were supported by 10.13039/100000935RSF project 19-75-10055, Numerical experiments were supported in the frames of the Governmental Project of the Institute of Applied Physics 10.13039/100011435RAS (Project #FFUF-2021-0014), In vivo experiments were supported by the Center of Excellence "Center of Photonics" funded by the 10.13039/501100004569Ministry of Science and Higher Education of the Russian Federation, Contract No. 075-15-2022-316. DR acknowledges support from the 10.13039/501100013362Swiss Cancer Research under grant KFS-5234-02-2021.

## Declaration of Competing Interest

The authors declare that they have no known competing financial interests or personal relationships that could have appeared to influence the work reported in this paper.

## Data Availability

Data is available upon reasonable request.
